# Association and mediation analyses among multiple metals exposure, plasma folate, and community-based impaired estimated glomerular filtration rate in central Taiwan

**DOI:** 10.1186/s12940-022-00855-x

**Published:** 2022-04-23

**Authors:** Mu-Chi Chung, Hui-Tsung Hsu, Yan-Chiao Mao, Chin-Ching Wu, Chih-Te Ho, Chiu-Shong Liu, Chi-Jung Chung

**Affiliations:** 1 Division of Nephrology, Department of Medicine, Taichung Veterans General Hospital, Taichung, Taiwan; 2 Department of Public Health, College of Public Health, China Medical University, No. 100, Sec. 1, Jingmao Rd., Beitun Dist, Taichung City, 406040 Taiwan; 3 Division of Clinical Toxicology, Department of Emergency Medicine, Taichung Veterans General Hospital, Taichung, Taiwan; 4 Department of Family Medicine, China Medical University Hospital, Taichung, Taiwan; 5 Department of Medical Research, China Medical University Hospital, Taichung, Taiwan

**Keywords:** Weighted quantile sum regression, Metals, Folate, Chronic kidney disease, Mediation analysis

## Abstract

**Background:**

Chronic kidney disease (CKD) is increasing, with heavy metal exposure an important risk factor. Additionally, the antioxidant folic acid has been studied for reducing blood arsenic levels and related tissue damage. Therefore, we explored the association and mediation effects among various heavy metal levels in blood, plasma folate, other CKD risk factors, and impaired estimated glomerular filtration rate (eGFR).

**Methods:**

We constructed a community-based cross-sectional study from the Human Biomonitoring and Environmental Health Program in central Taiwan. A total of 1643 participants had lived locally for > 5 years, > 40 years old, and completely received health examinations and biospecimen collections. Impaired eGFR was defined as one single eGFR < 60 mL/min/1.73 m^2^. Plasma folate and metal levels in blood were determined, as well as urinary 8-hydroxy-2′-deoxyguanosine as an oxidative stress marker. Generalized weighted quantile sum (WQS) regression analysis was used to calculate a WQS score, reflecting overall body-burden of multiple metals (arsenic, cadmium, chromium, nickel, and lead) in blood.

**Results:**

Impaired eGFR was identified in 225 participants. Participants with high WQS scores had increased risk of impaired eGFR (odds ratio = 1.67; 95% confidence interval [CI]: 1.34, 2.07). Of five metals, arsenic, lead, and cadmium were weighted highly in impaired eGFR. Participants with high WQS and folate insufficiency (< 6 ng/mL) had 2.38-fold risk of impaired eGFR compared to those with low WQS and high folate (≥6 ng/mL) (95% CI: 1.55, 5.17). Similar increased 4.16-fold risk of impaired eGFR was shown in participants with high WQS and uric acid levels (95% CI: 2.63, 6.58). However, there were no significant WQS–folate (*p* = 0.87) or WQS–uric acid (*p* = 0.38) interactions on impaired eGFR risk. As a mediator, uric acid contributed 24% of the association between WQS score and impaired eGFR risk (*p* < 0.0001). However, no mediation effect of plasma folate was observed.

**Conclusion:**

WQS analysis could be applied to evaluate the joint effects of multiple metals exposure. High WQS scores may influence impaired eGFR risk through increased uric acid levels. A large-scale and prospective cohort study is necessary to validate these results and demonstrate any causal relationship.

**Supplementary Information:**

The online version contains supplementary material available at 10.1186/s12940-022-00855-x.

## Introduction

Chronic kidney disease (CKD) is a growing public health issue globally with many known and unknown etiologies [1]. Some environmental factors have also been shown to be important risk factors for kidney injury [2]. Toxic metals, as common environmental pollutants, potentially increase the risk of CKD or accelerate its progression [2, 3].

Individual metal exposure has been indicated as associated with developmental nephrotoxicity in animal models [4] and with impaired kidney function in many epidemiological studies [3]. It is important to further explore multiple metals co-exposure with CKD risk because the joint effects may be synergistic or antagonistic. Previous studies pointed out multiple metals co-exposure is associated with additional decline in the eGFR (estimated glomerular filtration rate) [5] and increased risk of CKD [6] using multivariable model adjustment. However, using a highly correlated set of multiple metals exposure might result in collinearity and variance inflation in analysis of traditional regression models. Therefore, we applied weighted quantile sum (WQS) regression in our mixtures modeling approach in the present study. The WQS model is a recently developed statistical method to allow for a highly correlated set of metals to be considered in the model. It estimates a WQS score as connected with adverse health under maximizing a generalized linear function subject to constraints on the weights of each metal to sum to 1 [7, 8]. The WQS score reflects the body burden of metal mixtures. Furthermore, the important metals in the WQS scores can be identified by comparing the corresponding weight of individual metal contributions to the WQS scores [8, 9].

Folic acid metabolism is impaired in CKD patients [10]. Folic acid deficiency might be associated with progression of CKD through increased oxidative stress, decreased endothelial nitric oxide synthase and endothelial dysfunction [11]. In contrast, folic acid therapy delayed CKD progression in a large randomized clinical trial [12]. In addition, folate insufficiency may impede arsenic (As) methylation and thereby aggravate arsenic toxicity [13]. Particularly, low levels of folate from dietary intake or in plasma can modify the associations between blood lead (Pb) and high hyperhomocysteinemia, an established risk factor for chronic vascular diseases [14, 15]. However, correlations of the blood levels of metals and folate in plasma are not clearly known. Therefore, it is important to further explore the complex relationships among heavy metals, folate, and CKD risk.

In this study, general participants were recruited from the community and all received health examinations and biospecimen collections. First, we apply WQS regression to evaluate multiple metals co-exposure with the prevalent risk of baseline impaired eGFR. Next, the interaction between WQS scores and folate status on impaired eGFR is explored. Finally, we investigate potential mediators linking WQS scores to impaired eGFR for their presence and magnitude of mediation.

## Materials and methods

### Study design and participants

This was a community-based cross-sectional study. All study participants were recruited from the Human Biomonitoring and Environmental Health Program (HBEHP) in central Taiwan, including Taichung City, Changhua, and Nantou Counties. The aim of HBEHP is to explore the association of exposure to various persistent pollutants in the environment with human health risks, such as cardiovascular diseases and cancers. Residents who had lived locally for more than 5 years and aged over 40 years were invited to participate in the program through telephone and face-to-face interview for the baseline. After acquiring informed consent from all individuals, all volunteers were asked to receive health examinations and biospecimen collections in the cooperating clinics or hospitals. Among the initial voluntary participants (*N* = 2061), 86.5% (*n* = 1783), 85.4% (*n* = 1760), and 79.7% (*n* = 1643) agreed to the physical examination, completed the questionnaire interview, and provided blood as well as urine samples, respectively. The detail process of recruitment was shown in Supplement Fig. 1. The definition of impaired eGFR was based on a single moderate or established kidney function of eGFR level < 60 mL/min/1.73 m^2^, calculated using the Chronic Kidney Disease Epidemiology Collaboration (CKD-EPI) equation [16]. This resulted in 225 participants with impaired eGFR and 1418 without impaired eGFR. The study was approved by the Research Ethics Committee of China Medical University Hospital, Taichung, Taiwan.

### Collection of questionnaire information and biological specimens, and health examinations

At baseline, all analyzed variables regarding socio-demographic characteristics and lifestyle-related factors, such as cigarette smoking, as well as individual medical history were collected through face-to-face interviews with a structured questionnaire. Meanwhile, general health checkups and biochemistry examinations were performed, including baseline anthropometric, blood pressure, and plasma levels of triglycerides, total cholesterol, low-density lipoprotein cholesterol (LDL-C), high-density lipoprotein cholesterol (HDL-C), fasting plasma glucose, insulin, uric acid, and blood creatinine after an 8-h fasting period. We defined smoking status as nonsmokers and ever smokers (combining former smokers who had quit smoking at the time of recruitment and current smokers with more than 100 cigarettes in their lifetime). Cumulative cigarette smoking was further calculated as a summarization of multiplication of duration of cigarette smoking and pack of cigarettes per day. Individual medical histories including hypertension and Type 2 diabetes (yes/no) with clinical verifications were also collected. The TC/HDL ratio was considered as a marker for the presence of atherogenic dyslipidemia, and those with high cardiovascular risk were defined as a TC/HDL ratio ≥ 4 [16]. In addition, an abnormal level of uric acid was defined as ≥7 mg/dL. Homeostatic Model Assessment for Insulin Resistance (HOMA-IR) was used to measure insulin resistance (IR) and calculated as fasting plasma glucose (mg/dL) × insulin (mU/L)/405 [17].

### Measurement of heavy metals in blood and plasma folate

About 5–6 mL of blood was collected from each participant during recruitment for measurement of heavy metals and levels of folate in plasma. The plasma folate levels were measured using direct chemiluminescent technology according to the manufacturer’s instructions. Plasma folate insufficiency was defined as < 6 ng/ml [18]. All plasma samples were evaluated under dim yellow light. For replicate plasma samples, the mean coefficient of variation was within 10%.

The detailed protocol for determination of heavy metals in blood – including As, cadmium (Cd), chromium (Cr), nickel (Ni), and Pb – was previously described [19]. In brief, the multiple levels of the above metals in whole blood digested with nitric acid were quantified using inductively coupled plasma-mass spectrometry (ICP-MS; Agilent 7700c, Agilent Technologies, Inc., Palo Alto, CA, USA). Correlation coefficients for fitted calibration curves were ≥ 0.99, and each recovery rate was in the range of 85–115%. Standard reference materials (Seronorm™ Trace Elements Whole Blood) of blood heavy metals were used for quality control of the measurements. We divided the values by the square root of two, when the levels of blood heavy metals were less than the detection limit values: 0.03 ppb for As, 0.007 ppb for Cd, 0.054 ppb for Cr, 0.103 ppb for Ni, and 0.067 ppb for Pb. Detection frequencies of individual metals were ≥ 95%, except for Ni with 92% (Supplementary Table 1). For experimental reliability, repeated samples were randomly selected and analyzed. Coefficients of variation were within the range of 5–10% for each metal.

### Urinary 8-hydroxy-2′-deoxyguanosine (8-OHdG) determination

Urinary levels of 8-OHdG, commonly considered a reliable and abundant marker reflecting the degree of oxidative stress [20, 21], were determined by online automated online solid-phase extraction coupled with liquid chromatography – electrospray tandem mass spectrometry (API3000, Applied Biosystems, MDS SCIEX, Concord, ON, Canada), according to a published protocol [22]. The calibration curves for detection of urinary 8-OHdG had good linearity with R^2^ > 99% and detection limits were 10 pg/mL. The accuracy of intra- and inter-day measurements was 100 ± 15%.

### Statistical analysis

Descriptive data are shown as mean and standard deviation for continuous variables and number and percentage for categorical variables. We first used age- and sex-adjusted logistic regression models to calculate the odds ratio (OR) and 95% confidence interval (95% CI) and to investigate the associations among lifestyle-related factors, clinical comorbidities, Homeostatic Model Assessment for Insulin Resistance (HOMA-IR), plasma folate, and urinary 8-OHdG as well with impaired eGFR risk. Additionally, we used single-pollutant models of multiple logistic regressions, with adjustment for potential risk factors (e.g. age, sex, BMI, type 2 diabetes, and hypertension) to explore the association between individual metals and impaired eGFR risk. Multi-pollutant models were further executed when considering all other heavy metals.

For WQS regression analysis as shown in Eq. (1) [8], continuous levels of metals in blood were categorized into quartiles (q_i_ = 0, 1, 2, and 3 indicating the 1st, 2nd, 3rd, and 4th quartile, respectively) based on examination of the distribution of residuals for normality in models [23].

1 $$\log \left[\mathrm{P}\left(\mathrm{Y}=1\right)/\mathrm{P}\left(\mathrm{Y}=0\right)\right]={\upbeta}_0+{\upbeta}_1\left(\sum \limits_{i=1}^{\mathrm{c}}{\mathrm{w}}_{\mathrm{i}}{\mathrm{q}}_{\mathrm{i}}\right)+\mathrm{z}\hbox{'}\upphi,$$

2 $$WQS=\sum \limits_{i=1}^c{w}_i{q}_i,$$

where Y represents the binary outcome of impaired eGFR in Eq. (1), β_0_ is the intercept, β_1_ is the regression coefficient of WQS scores, z is a vector of covariates with adjustment in the model, and ϕ is a vector of regression coefficients for the covariates. Meanwhile, w_i_ is the weight for the ith metal. In addition, because WQS regression provides a unidirectional evaluation of mixture effects, we set β_1_ as a positive coefficient without constraint. Average weights were determined across 10,000 bootstrapped samples under maximization of the log likelihood of impaired eGFR risk. Each weight of five metals was constrained between 0 and 1 with sum to 1. The WQS score as shown in Eq. (2) was defined as the weighted index for the set of five metals and reflected the whole-body burden of five metals. Furthermore, WQS regression analysis identified the important metals among the multiple metals by comparing the contributions of the empirically estimated weights to the WQS scores [8]. The WQS analysis was performed using the gWQS package in R version 3.6.3. We further evaluated the association of WQS scores with impaired eGFR risk after adjusting for age, sex, BMI, type 2 diabetes, and hypertension. We compared the relationships between WQS scores and log-transformed levels of folate, uric acid, HOMA-IR, 8-OHdG, and TC/HDL ratio in individuals through univariate linear regression models. Multiplicative and additive interactions among WQS scores, folate, and uric acid with impaired eGFR risks were further evaluated using multiple logistic regressions. We observed the product terms of the two desired factors on the impaired eGFR risk in a logistic regression model to test for multiplicative interaction. For additive interactions, we calculated the index of relative excess risk due to interaction (RERI) as OR_11_ − OR_10_ − OR_01_ + 1, where OR _11_ is the OR of impaired eGFR for people with high WQS scores but low levels of plasma folate, OR_10_ is the OR of impaired eGFR for people with high WQS scores and high levels of plasma folate, and OR_01_ is the OR of impaired eGFR for people with low WQS scores and low levels of plasma folate. The RERI > 0 indicates a positive interaction and the 95%CI does not include 0. Finally, we performed a causal mediation analysis to evaluate the direct effect of WQS scores on impaired eGFR risk independent of a mediator (e.g. folate, uric acid, HOMA-IR, 8-OHdG, and TC/HDL ratio) [24]. The roles that the mediators played in the indirect effect as well as the percentages mediated (adjusted for confounding variables) were also calculated. Also, a binary outcome and a continuous mediator were used in the mediation analysis with no statistically significant cross-product interaction of exposure and mediator; the expressions were simply reduced to (*e*_*1*_ – *e*_*0*_)*θ*_E_ and (*e*_*1*_ – *e*_*0*_)*β*_M_*θ*_E_ for comparing WQS scores per increased unit (*e*_*1*_ – *e*_*0*_) for direct and indirect effects, respectively. Also, a proportion-mediated percentage was calculated from the direct (DE) and indirect effect (IE) odds ratios using the formula OR^DE^(OR^IE^ − 1)/(OR^DE^OR^IE^ − 1) [24]. All data were analyzed using the SAS statistical package (SAS, version 9.4, Cary, NC, USA) or R software version 3.6.3 (The R Foundation for Statistical Computing; Vienna, Austria). A two-sided *p*-value < 0.05 was considered significant.

## Results

### Descriptive characteristics, plasma folate, and impaired eGFR risk

Based on the CKD-EPI equation for renal function evaluation, we screened 225 participants with eGFR < 60 mL/min/1.73 m^2^ from the total population (*N* = 1643). In Table 1, we compared the baseline characteristics, lifestyle-related variables, and biochemistry values between impaired and non-impaired eGFR groups. The impaired eGFR group was more likely to be higher age, female, overweight and obese, and of lower education level, with a history of hypertension and Type 2 diabetes compared to controls. For biochemistry values, the estimated ORs of impaired eGFR significantly increased per unit increment for uric acid (*p* < 0.0001), HOMA-IR (*p* < 0.0001), and TC/HDL ratio (*p* = 0.0245) after adjusting for age and sex. There were similar levels of plasma folate between impaired and non-impaired eGFR groups. However, participants with folate insufficiency (< 6 ng/mL) had 1.54 increased odds of impaired eGFR (95%CI: 1.09, 2.17).

**Table 1:** Descriptive characteristics between study participants with impaired and non-impaired eGFR groups

Variables	Impaired eGFR	Non-impaired eGFR	Age and sex-adjusted ORs^a^ (95%CI)
	*n* = 225	*n* = 1418	
Age, median (IQR), year	73 (13)	61 (17)	1.09 (1.07, 1.11) ^***^
Male (%)	63 (28.00)	689 (48.59)	1.88 (1.36, 2.60) ^***^
BMI, median (IQR), kg/m^2^	24.72 (4.11)	24.14 (4.12)	
< 18.5	12 (5.33)	55 (3.88)	2.34 (1.11, 4.93) ^*^
18.5–24	70 (31.11)	644 (45.42)	Reference
24–27	83 (36.89)	469 (33.07)	1.62 (1.13, 2.33) ^**^
> = 27	60 (26.67)	250 (17.63)	2.64 (1.76, 3.97) ^***^
Education			
Elementary school or lower	83 (37.22)	321 (22.75)	Reference
High school	89 (39.91)	625 (44.29)	0.98 (0.68, 1.40)
College or higher	51 (22.87)	465 (32.96)	0.73 (0.48, 1.11)
Smokers (%)	1103 (45.78)	480 (33.85)	1.19 (0.86, 1.65)
Secondhand smoking exposure (%)	69 (30.67)	524 (37.16)	0.92 (0.67, 1.28)
Alcohol drinkers (%)	76 (33.78)	429 (30.25)	0.83 (0.59, 1.17)
Hypertension	135 (60.00)	413 (29.13)	2.21 (1.62, 3.01) ^***^
Type 2 Diabetes	49 (21.78)	122 (8.60)	2.45 (1.65, 3.63) ^***^
Uric acid, median (IQR), mg/dL	6.10 (2.40)	5.30 (1.80)	1.48 (1.33, 1.66) ^***^
< 7	148 (66.07)	1231 (87.43)	Reference
> =7	76 (33.93)	177 (12.57)	3.20 (2.24, 4.56) ^***^
Insulin, median (IQR), μU/mL	10.05 (10.70)	7.30 (6.70)	1.03 (1.02, 1.03) ^***^
HOMA-IR, median (IQR)	2.42 (3.30)	1.66 (1.88)	1.06 (1.04, 1.08) ^***^
< 1.9	80 (35.71)	798 (56.68)	Reference
> =1.9	144 (64.29)	610 (43.32)	2.20 (1.61, 3.00) ^***^
TC/HDL ratio, median (IQR)	4.43 (1.35)	4.13 (1.44)	1.20 (1.02, 1.40) ^*^
< 4	165 (73.66)	1088 (76.94)	Reference
> =4	59 (26.34)	326 (23.06)	1.06 (0.75, 1.51)
Folate in plasma, median (IQR), ng/mL	8.68 (9.36)	9.79 (8.20)	0.98 (0.96, 1.01)
< 6	67 (29.78)	302 (21.30)	1.54 (1.09, 2.17) ^***^
> =6	158 (70.22)	1116 (78.70)	Reference
8-OHdG, median (IQR), ng/mg creatinine	4.45 (3.57)	4.89 (3.97)	1.02 (0.98, 1.05)

*IQR* interquartile range. ^a^ Odds ratios (ORs) and 95% confidence interval (95%CI) for risk of CKD were calculated using multivariate logistic regression model. ^*^ 0.01 < *p* < 0.05; ^**^ 0.001 < *p* < 0.01; ^***^ *p* < 0.001

### Association between blood levels of five metals and impaired eGFR risk

Median (interquartile range) of individual metals was 4.93 (4.13) μg/L for As, 0.76 (0.58) μg/L for Cd, 0.15 (2.25) μg/L for Cr, 1.35 (3.14) μg/L for Ni, and 2.07 (1.25) μg/dL for Pb. We present the association between individual metals with impaired eGFR risk in the single-pollutant analysis in Table 2. The similar results showed that high levels of As and Pb were positively associated with increased OR of impaired eGFR both in the crude model and in multivariate logistic regression models. For multi-pollutant analysis, high levels of As in blood were similarly associated with impaired eGFR risk after considering the effects of other metals (Cd, Cr, Ni, and Pb).

**Table 2:** Comparisons of associations between urinary heavy metals and risk of impaired eGFR under single- and multi-pollutant models

Metals in blood	Crude ORs (95% CI) (single-pollutant)	ORs (95% CI) ^a^ (single-pollutant)	ORs (95% CI) ^b^ (multi-pollutant)
As, μg/L	1.06 (1.03, 1.09) ^***^	1.05 (1.02, 1.08) ^***^	1.05 (1.02, 1.08) ^***^
Cd, μg/L	1.03 (0.80, 1.32)	1.17 (0.89, 1.52)	1.07 (0.80, 1.43)
Cr, μg/L	1.00 (0.97, 1.04)	1.01 (0.98, 1.05)	1.01 (0.97, 1.06)
Ni, μg/L	0.98 (0.94, 1.02)	0.99 (0.95, 1.03)	0.98 (0.93, 1.03)
Pb, μg/dL	1.02 (1.01, 1.03) ^**^	1.02 (1.00, 1.03) ^*^	1.01 (1.00, 1.03) ^#^

^#^0.05 < *p* < 0.1; ^*^ 0.01 < *p* < 0.05; ^**^ 0.001 < *p* < 0.01; ^***^ *p* < 0.001

SD: standard deviation; IQR: interquartile range

^a^ Models adjusted for age, sex, BMI (category), diabetes, and hypertension

^b^ Models adjusted for age, sex, BMI (category), diabetes, hypertension, and all metals

**Fig. 1:**
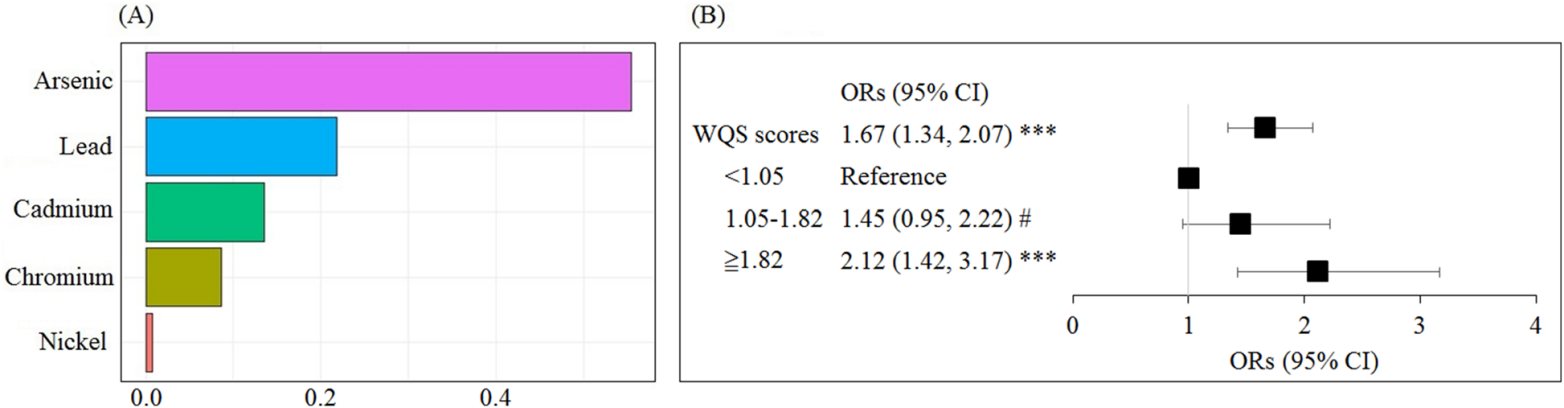
Relationships of WQS scores and impaired eGFR risk. **A** Relative weight values of As, Cd, Cr, Ni, and Pb in blood used in calculation of WQS scores. **B** Relationship of WQS scores and impaired eGFR risk. WQS scores of the controls were set as the cut-off values. All models were adjusted for age, sex, BMI (category), diabetes, and hypertension

**Fig. 2:**
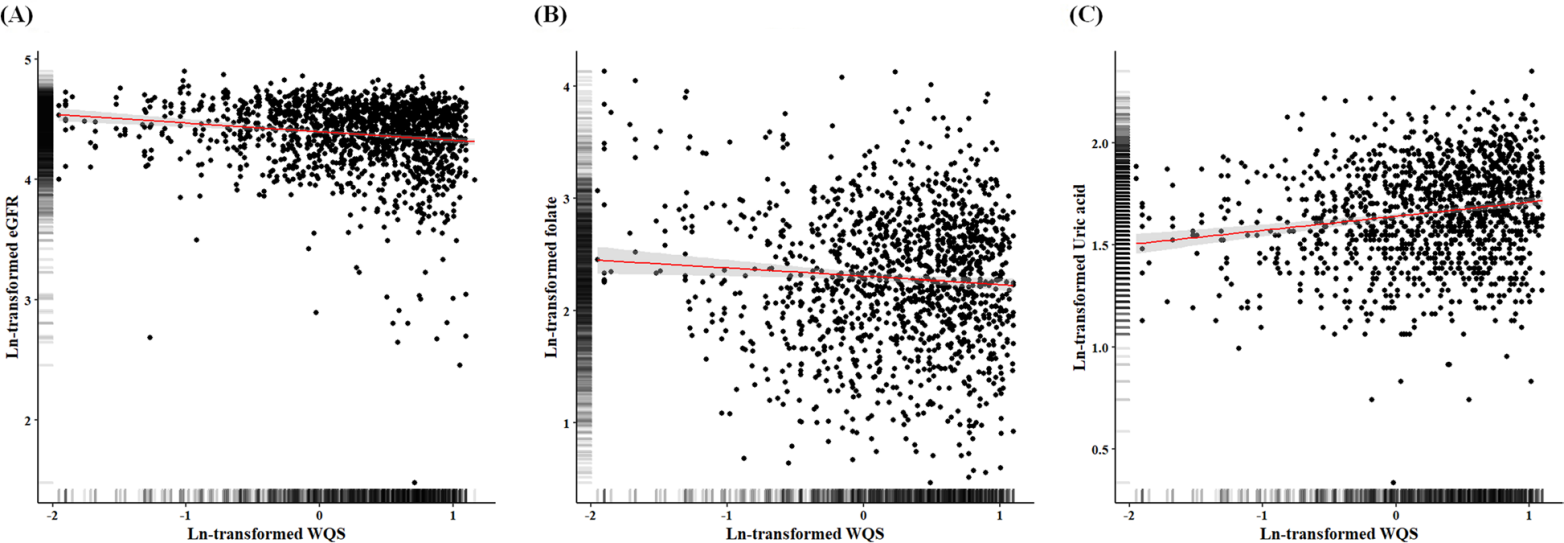
Correlations between (**A**) WQS scores and eGFR, (**B**) WQS scores and folate, and (**C**) WQS scores and uric acid. All values presented were natural log-transformed

We then analyzed the association between WQS scores of metal mixtures and the risk of impaired eGFR. In the WQS analysis, the weightings of five metals were as follow: As (55.4%), Pb (21.8%), Cd (13.5%), Cr (8.6%), and Ni (0.7%) (Fig. 1A). A 1.67-fold increased risk of impaired eGFR (95%CI: 1.34, 2.07, *p* < 0.0001) was observed for per unit increment of WQS scores. Furthermore, to determine the dose–response relationship of WQS scores and impaired eGFR, we divided the WQS scores into tertile based on the 33.33th and 66.67th cutoffs of WQS scores of non-impaired eGFR groups (Fig. 1B). The results demonstrated that participants with WQS scores ≥1.82 had increased odds of impaired eGFR compared to those with < 1.05 (OR = 2.12; 95% CI: 1.42, 3.17). A significant dose–response relationship of WQS scores and impaired eGFR risk was observed (trend *p* < 0.0001). Also, the negative relationship of WQS scores with continuous eGFR is shown in Fig. 2A (β = − 2.53, *p* < 0.0001) after adjusting for age, sex, BMI, type 2 diabetes, and hypertension.

### Relationships of WQS scores, uric acid, and plasma folate with impaired eGFR risk

After natural logarithm transformations of WQS scores, plasma folate, uric acid, and other biochemistry values, we determined the relationships among these variables in the control group through multiple linear regression analysis. There was a negative association between WQS scores and plasma folate (β = − 0.07, *p* = 0.007; Fig. 2B) as well as a positive association between WQS scores and uric acid (β = 0.05, *p* < 0.0001; Fig. 2C). However, no correlations between WQS scores, and other biochemistry values were observed.

We determined the median cutoff based on the WQS scores of the non-impaired eGFR group and explored the interactions of WQS-folate and WQS-uric acid on risk of impaired eGFR (Table 3). Participants with both high WQS and low levels of folate had the highest risk of impaired eGFR compared to those with low WQS and high levels of folate (OR = 2.83; 95%CI: 1.55, 5.17) after adjusting by age, sex, BMI (category), diabetes, and hypertension. Similarly, participants with both high WQS and high levels of uric acid had the highest risk of impaired eGFR (OR = 4.16; 95%CI: 2.63, 6.58). We further evaluated the multiplicative or additive interaction using cross-product terms or RERI in the analysis. However, no significant interactions of WQS scores either with plasma folate or uric acid were shown. Finally, we determined uric acid and plasma folate as potential mediators of the association between WQS score and impaired eGFR risk. The mediator of uric acid contributed about 24% in the association between WQS score and impaired eGFR risk (*p* < 0.0001). However, there were no significant mediations by plasma folate in the relationship between WQS score and impaired eGFR risk.

**Table 3:** Direct and indirect effects of WQS scores on impaired eGFR risk with the levels of plasma folate and uric acid as the mediators

		Case/control number	OR (95%CI)	
WQS scores	Plasma folate			
< 1.41	≥ 6	56/546	1.00^&^	
< 1.41	< 6	25/163	1.51 (0.76, 3.01)	
≥ 1.41	≥ 6	102/570	2.01 (1.28, 3.17) ^**^	
≥ 1.41	< 6	42/139	2.83 (1.55, 5.17) ^***^	
			trend *p* = 0.0002	
			multiplicative interaction *p* = 0.8664	
			RERI (95% CI) = 0.35 (−0.82, 1.52)	
WQS scores	Uric acid			
< 1.41	< 7	63/640	1.00	
< 1.41	> = 7	17/64	2.17 (1.12, 4.22) ^*^	
≥1.41	< 7	85/591	1.35 (0.93, 1.96)	
≥1.41	> = 7	59/113	4.16 (2.63, 6.58) ^***^	
			trend *p* < 0.0001	
			multiplicative interaction *p* = 0.3824	
			RERI (95% CI) = 1.64 (−0.36, 3.64)	
Mediator:		NDE (95%CI)	NIE (95%CI)	PM (%) (95%CI)
Plasma folate		1.65 (1.29, 2.01) ^***^	1.00 (0.99, 1.01)	−0.21 (−1.31, 0.88)
Uric acid		1.51 (1.18, 1.85) ^**^	1.11 (1.06, 1.16) ^***^	24.07 (11.69, 36.45) ^***^

*RERI* relative excess risk due to interaction, *NDE* Natural direct effect, *NIE* Natural indirect effect, *PM* Estimated proportion mediated

All models adjusted for age, sex, BMI (category), diabetes, and hypertension. ^#^0.05 < *p* < 0.1; ^*^ 0.01 < *p* < 0.05; ^**^ 0.001 < *p* < 0.01; ^***^ *p* < 0.001

## Discussion

This is the first study to evaluate WQS scores, reflecting overall body burden of metal mixtures and further explore the potential mediators involved in the association of exposure to environmental heavy metals with impaired eGFR risk. The results indicated a significant dose–response relationship between WQS scores and increased risk of impaired eGFR. Participants with high WQS scores and either folate insufficiency or high levels of uric acid had increased risk of impaired eGFR. Further we observed a significant mediated proportion of CKD risk of about 24% for uric acid.

In this study, we included the hazardous metals As, Pb, Cd, Cr, and Ni, because some of them are globally known as nephrotoxic metals [3, 4]. Consistent positive association between As exposure and albuminuria, CKD, and further kidney disease mortality was reported in a previous systemic review [25]. One prospective study in Taiwan found every increase of 1 mg/dL in blood Pb level at baseline was associated with a decrease in GFR of 4.0 mL/min/1.73 m^2^ after 4 years [26]. Several studies also reported higher blood Cd level was associated with decreased glomerular filtration and increased urine protein excretion [6, 27]. The possible mechanisms of exposure to various metals involved with prevalence of CKD and eGFR annual decline were discussed in previous studies [3]. Fewer reports have considered the association between blood Cr or Ni and renal function. One report in Taiwan found that doubling of urinary Cr decreased eGFR by 5.99 mL/min/1.73 m^2^ [28].

The individual levels of heavy metals in our participants were under the limits for acute effect doses; therefore, to explore the joint effect of metal mixtures is important to understand CKD development. Previous studies found that Pb and Cd co-exposure was a stronger determinant of renal injury biomarkers [29] and CKD [6]. Another study also reported co-exposure to Cr with Pb and Cd is associated with additional eGFR decline [28]. These interactive toxic effects can be explained by the synergistic effect of heavy metals targeting renal tubule injury, proximal tubular atrophy associated with interstitial fibrosis, increased reactive oxygen species, and inducing oxidative stress in the kidney [4]. However, few studies have explored co-exposure of three and more kinds of heavy metals on renal function using WQS analysis. One study found WQS models using the combined blood metals (Cd, Pb, and Hg) were associated with adverse effects on multiple renal parameters in adolescents [23]. The WQS was recently developed to analyze the effects of complex exposures and quantify the joint effect of mixtures on health. In addition, this approach weights the contribution of individual mixture components and is possibly more sensitive than single-chemical analyses in identifying important factors [30]. In our study, As and Pb were significantly associated with impaired eGFR in multiple logistic regressions. In further WQS regression analysis, As (55.4%), Pb (21.8%), and Cd (13.5%) accounted for a notable proportion of the risk of impaired eGFR (Fig. 1). Of these, Cd showed no significant association with impaired eGFR in the generalized linear regression model. This suggests a high correlation of urinary Pb and Cd in our participants. In real life, people are exposed to all of these five metals simultaneously at different levels. Detection frequency of the five metals exceeded 90% in our study population. In WQS analysis, As was weighted highly, but the risk of impaired eGFR was low with exposure to low As. Therefore, the WQS score reflects the overall exposure to all the metals and it is difficult to elucidate the interaction between any two of these metals [30].

There are conflicting findings of plasma folate insufficiency with CKD risk in the present results and previous studies [31, 32]. This might be explained by the status of folate insufficiency in Taiwan. There is no fortification policy in Taiwan and the trend in folate insufficiency has not improved in recent years, even worsening in the young population [18]. In our study, people with folate insufficiency combined with high score of metal-related WQS were associated with a higher risk of CKD. Given the critical role of folate in one-carbon metabolism pathway as the donor of S-adenosylmethionine, folate insufficiency may impede As methylation and thereby exacerbate As toxicity [13, 33]. One randomized controlled trial showed that folate supplementation (at least 400 μg per day) may lower total As concentrations in blood through increasing urinary excretion of As, including decrease in percentages of inorganic As and monomethyl-As species as well as increase in percentage of dimethyl-As species [13]. An inverse relationship between plasma folate levels and blood Pb levels has also been reported [34]. In addition, CKD possibly reduced expressions of folate transporters in multiple organs and further impaired cellular homeostasis of these essential micronutrients [35]. Due to its beneficial role as an antioxidant, plasma folate levels should be considered when considering issues of heavy metal toxicity [36].

We found a positive association between hyperuricemia and risk of impaired eGFR. Abundant evidence has indicated that hyperuricemia independently predicts the incidence and development of CKD through endothelial dysfunction, activation of the renin–angiotensin system, and enhances oxidative stress within the cell [37]. Interestingly, we found that co-exposure of hyperuricemia and high combined blood metal WQS score were associated with higher impaired eGFR risk; in addition, the association of combined blood metals with impaired eGFR risk was partly mediated by hyperuricemia. A previous study revealed that As exposure may be associated with hyperuricemia risk in men and with gout prevalence in women. Exposure to As could result in hyperuricemia secondary to kidney injury in animal studies [38]. Furthermore, elevated Pb exposure is also a well-defined risk factor for hyperuricemia and gout [39], possibly through increased production or decreased excretion of uric acid. Indeed, we found a positive relationship between combined blood metal WQS score and blood urate levels. Toxicity of heavy metals themselves and hyperuricemia induced by heavy metal exposure possibly explain the increased risk of impaired eGFR. Furthermore, there is a significant association between hyperhomocysteinemia and hyperuricemia in metabolic syndrome [40], and folate has been reported to potently inhibit xanthine oxidase [41]. Folic acid therapy reduced serum uric acid in hypertensive patients in a large randomized clinical trial [42]. The complex mechanisms related to heavy metals, folate, and uric acid could not be clarified in our cross-sectional study and deserve further investigation.

Our study had a number of strengths. We assessed the relationship between multiple metal co-exposures by WQS regression analysis and kidney function in a large, community-based population with collected exposure and other risk factors of impaired eGFR. This study also demonstrated the interactions among metal co-exposures, folate insufficiency, and hyperuricemia for impaired eGFR risk, which was rarely mentioned previously. However, some limitations of our study need to be considered. First, one time-point of blood metals, plasma folate, and uric acid was analyzed in the cross-sectional study design. This prevents any inference of a temporal relationship of exposure, mediator, and outcome. For mediation analysis, there were essential assumptions should be considered, including control for all confounding from exposure-outcome, exposure-mediator and mediator-outcome as well as no mediator-outcome confounder affected by the exposure [24]. Therefore, a prospective time-course study of a large population is needed in future studies to validate the findings of the present study. In addition, we adopted the five metals in the same medium of blood in the WQS analysis. We did not explore the roles of urinary As species in CKD such as monomethyl-As as well as dimethyl-As species, which are regarded as the main metabolites of As. However, based on findings of a previous randomized controlled trial, folic acid supplementation may lower the total levels of As in blood with increased methylation of inorganic As to dimethyl-As species [13]. Hence, the blood As levels might be a surrogate index of the body burden. Second, levels of plasma folate or urinary 8-OHdG may be modified by dietary habits or intake of vitamin supplements. No detailed information on dietary factors was acquired, which may result in no associations between WQS and plasma folate (or urinary 8-OHdG) in our analysis. Also, measurements of serum folate are not very indicative of tissue folate status, and we made no red blood cell folate measures. Further, the assumption that all metals had the same effect direction (either positive or negative) for the impaired eGFR association was constrained in the WQS regression model [8]. Previous literature elucidated the issue of the reversal paradox in different processes of inference for WQS analysis [9, 43, 44]. In the present study, we chose five hazardous metals for the WQS analysis to avoid the reduced accuracy of associations and to improve the interpretability of the weighted index [45]. In addition, the magnitude of exposure might be lowered when replacing continuous levels of individual metals with quantile variables in the model. However, compared with traditional regression, WQS regression has more sensitivity and specificity for the inference of the weighted index and indicating the important components of the metal mixtures [46]. We merely explored five hazardous metals in the WQS analysis, which could exclude effects of exposure to other metals. Finally, the results may not be fully representative of the Taiwanese general population.

## Conclusion

We executed generalized WQS regression analysis and considered both the weights and the doses of multiple metals to acquire the WQS scores, which reflect overall body burden of exposure to multiple metals. Community participants high WQS scores and either folate deficiency or high levels of uric acid had increased 2.83–4.16-fold risk of impaired eGFR. Uric acid mediated of significant proportion of about 24% of the impaired eGFR risk. In the future, a large cohort study will be necessary to validate these results and further demonstrate any causal relationship.

## Supplementary Information

**Additional file 1: Supplementary Figure 1.** Flow chart showing study participants selection.

**Additional file 2: Supplementary Table 1.** Detection frequency and distributions of 5 heavy metals in blood.

## Data Availability

Data used and analyzed in the manuscript will be made available upon request.

## Abbreviations

CKD: Chronic kidney disease
eGFR: Estimated Glomerular filtration rate
WQS: Weighted quantile sum
