# Supplementary figures and images for: Association and mediation analyses among multiple metals exposure, plasma folate, and community-based impaired estimated glomerular filtration rate in central Taiwan
Source: Environ Health. 2022 Apr 23;21:44. doi: 10.1186/s12940-022-00855-x (PMC9034511; doi:10.1186/s12940-022-00855-x)

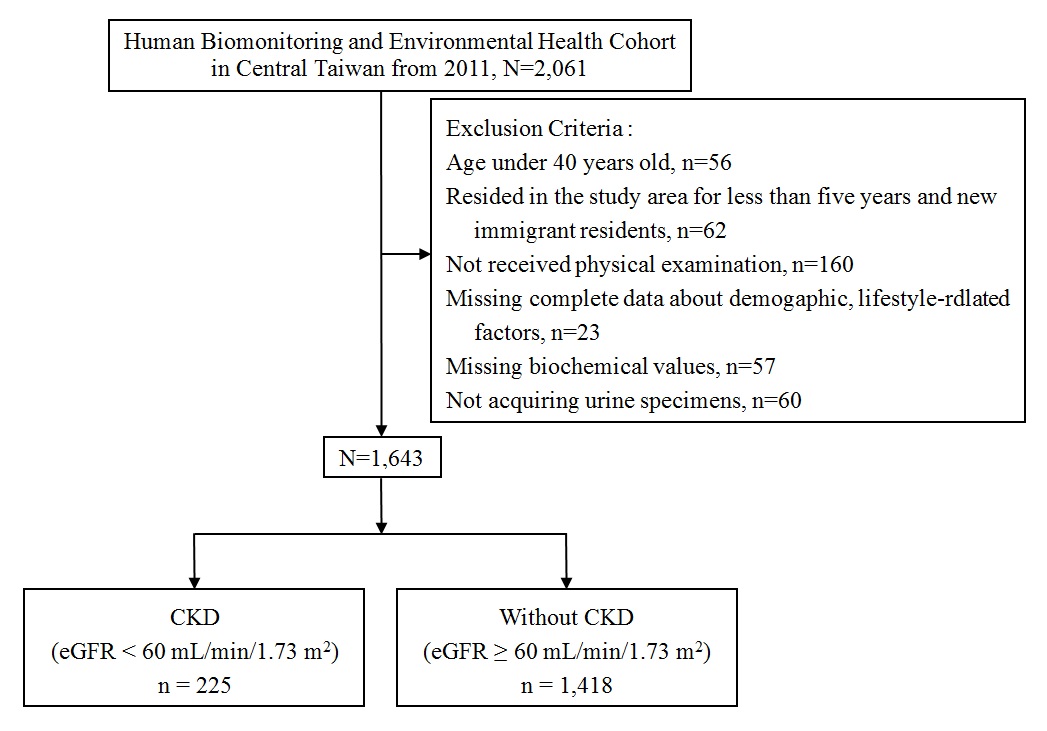

Supplement: Supplementary file 1 — Additional file 1: Supplementary Figure 1. Flow chart showing study participants selection. [file 12940_2022_855_MOESM1_ESM.jpg]
