# Supplementary material for: Association and mediation analyses among multiple metals exposure, plasma folate, and community-based impaired estimated glomerular filtration rate in central Taiwan
Source: Environ Health. 2022 Apr 23;21:44. doi: 10.1186/s12940-022-00855-x (PMC9034511; doi:10.1186/s12940-022-00855-x)
Supplement: Supplementary file 2 — Additional file 2: Supplementary Table 1. Detection frequency and distributions of 5 heavy metals in blood. [file 12940_2022_855_MOESM2_ESM.docx]

Supplementary Table 1. Detection frequency and distributions of 5 heavy metals in blood

|  | | LOQ | DF (%) | Mean (SD) | Median (IQR) |
| --- | --- | --- | --- | --- | --- |
| As (μg/L) |  | 0.030 | 100 | 5.85 (4.53) | 4.93 (4.13) |
| Cd (μg/L) |  | 0.007 | 100 | 0.85 (0.56) | 0.76 (0.58) |
| Cr (μg/L) |  | 0.054 | 95 | 2.03 (4.34) | 0.15 (2.25) |
| Ni (μg/L) |  | 0.103 | 92 | 2.66 (4.13) | 1.35 (3.14) |
| Pb (μg/dL) |  | 0.067 | 100 | 2.29 (1.14) | 2.07 (1.25) |

DF: detection frequency (%)
